# Alternative splicing: hallmark and therapeutic opportunity in metabolic liver disease

**DOI:** 10.1093/gastro/goaf044

**Published:** 2025-05-27

**Authors:** Mingqian Jiang, Saleh A Alqahtani, Wai-Kay Seto, Yusuf Yilmaz, Ziyan Pan, Luca Valenti, Mohammed Eslam

**Affiliations:** Department of Endocrinology and Metabolism, People’s Hospital Affiliated to Fujian University of Traditional Chinese Medicine, Fuzhou, Fujian, P. R. China; Storr Liver Centre, Westmead Institute for Medical Research, Westmead Hospital and University of Sydney, NSW, Australia; Liver, Digestive, & Lifestyle Health Research Section, and Organ Transplant Center of Excellence, King Faisal Specialist Hospital & Research Center, Riyadh, Saudi Arabia; Division of Gastroenterology and Hepatology, Weill Cornell Medicine, New York, NY, USA; Department of Medicine, The University of Hong Kong, Hong Kong, P. R. China; State Key Laboratory of Liver Research, The University of Hong Kong, Hong Kong, P. R. China; Department of Gastroenterology, School of Medicine, Recep Tayyip Erdoğan University, Rize, Türkiye; Storr Liver Centre, Westmead Institute for Medical Research, Westmead Hospital and University of Sydney, NSW, Australia; Department of Pathophysiology and Transplantation, Università degli Studi di Milano, Milan, Italy; Precision Medicine, Biological Resource Center, Fondazione IRCCS Ca’ Granda Ospedale Maggiore Policlinico Milano, Milan, Italy; Storr Liver Centre, Westmead Institute for Medical Research, Westmead Hospital and University of Sydney, NSW, Australia

**Keywords:** alternative splicing, metabolic dysfunction, liver disease, fibrosis, antisense oligonucleotides

## Abstract

Metabolic dysfunction-associated fatty liver disease (MAFLD) has become the leading cause of chronic liver disease worldwide, with fibrosis recognized as the main prognostic factor and therapeutic target. While early-stage fibrosis is reversible, advanced fibrosis poses a significant clinical challenge due to limited treatment options, highlighting the need for innovative management strategies. Recent studies have shown that alternative pre-mRNA splicing, a critical mechanism regulating gene expression and protein diversity, plays a fundamental role in the pathogenesis of MAFLD and associated fibrosis. Understanding the complex relationship between alternative splicing and fibrosis progression in MAFLD could pave the way for novel therapeutic approaches and improve clinical outcomes. In this review, we describe the intricate mechanisms of alternative splicing in fibrosis associated with MAFLD. Specifically, we explored the pivotal of splicing factors, and RNA-binding proteins, highlighting their critical interactions with metabolic and epigenetic regulators. Furthermore, we provide an overview of the latest advancements in splicing-based therapeutic strategies and biomarker development. Particular emphasis is placed on the potential application of antisense oligonucleotides for rectifying splicing anomalies, thereby laying the foundation for precision medicine approaches in the treatment of MAFLD-associated fibrosis.

## Introduction

Metabolic dysfunction-associated fatty liver disease (MAFLD) also known as metabolic dysfunction-associated steatotic liver disease (MASLD)–formerly known as nonalcoholic fatty liver disease (NAFLD)–has emerged as the predominant cause of chronic liver disease, impacting over a quarter of the global adult population [1–7]. This condition can progress from simple steatosis to more severe forms, including steatohepatitis, liver fibrosis, cirrhosis, and even hepatocellular carcinoma (HCC). The presence of fibrosis in MAFLD is a critical determinant of patient prognosis, serving as the most significant predictor of long-term outcomes such as liver-related complications and mortality [8, 9]. In general, MAFLD-associated fibrosis can be conceptualized as a complex process driven by a dynamic interplay of genetic, epigenetic, and environmental factors.

From a clinical standpoint, the presentation of MAFLD is highly heterogeneous, influenced by a complex interplay of genetic predisposition, lifestyle factors, coexisting metabolic disorders, and environmental exposures. These variables contribute to significant variability in disease progression, as well as differences in treatment response [10–12]. In addition, there are substantial interindividual differences in the development and progression of MAFLD-associated fibrosis. Accordingly, approximately 20% of patients with metabolic dysfunction-associated steatohepatitis (MASH), formerly nonalcoholic steatohepatitis (NASH), are classified as “rapid progressors”. While the precise predictors of rapid progression remain unclear, genetic susceptibility is believed to play a significant role in influencing disease trajectory [13].

The U.S. Food and Drug Administration (FDA) recently approved Resmetirom (Rezdiffra)–a thyroid hormone receptor-β agonist–for the treatment of adults with noncirrhotic NASH/MASH accompanied by significant liver fibrosis [14]. This approval marks a significant milestone as it is the first therapy approved for NASH/MASH, offering a new treatment avenue for patients with noncirrhotic NASH and advanced fibrosis. Despite the ongoing investigation of emerging therapies that target specific metabolic pathways and fibrosis in MAFLD, the drug development process for this condition continues to pose substantial challenges.

Currently, a major focus of research in MAFLD is transcriptional regulation, emphasizing the impact of gene upregulation and downregulation on disease progression. In addition, post-transcriptional regulation, particularly alternative RNA splicing, plays a pivotal yet underexplored role in MAFLD pathogenesis. Notably, aberrant splicing patterns can contribute to disease progression by generating protein variants that drive key pathological features, such as steatosis, inflammation, and fibrosis [15]. Importantly, the recent development of new technologies such as long-read sequencing enabling the direct detection and discovery of whole transcripts, quantification and their quantification, including at the single cell level [16], holds the promise to revolutionize the field in the near future enabling to uncover a new layer of regulation of biological pathways.

In this review, we present an updated overview of RNA splicing regulation in MAFLD-associated fibrosis, with the aim of uncovering novel insights that could inform the development of innovative therapeutic strategies.

## RNA splicing

**Figure 1.**
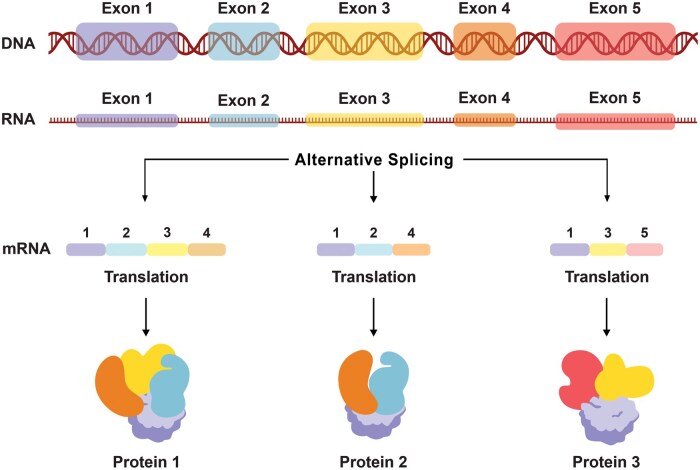
Overview of the alternative splicing process.

RNA splicing is an essential step in the maturation of messenger RNA (mRNA) and other RNA species, where precursor mRNA (pre-mRNA) is processed to form mature mRNA by excising noncoding regions (introns) and ligating coding regions (exons) [17, 18]. Notably, alternative splicing–a regulatory mechanism of RNA splicing–allows a single gene to generate multiple mRNA variants by selectively including or excluding different exons, thereby increasing the functional complexity of the genome [19]. Conversely, the concept of a “splicing switch” refers to alterations in the splicing process, carried out by the spliceosome, that lead to the inclusion or exclusion of specific exons, or the retention of introns, producing different protein isoforms from the same gene. This process relies on specific sequences at the 5' and 3' splice sites, which define the exon–intron junctions, and is influenced by various regulatory factors, such as RNA-binding proteins. These isoforms can have diverse and even opposing biological functions [20, 21], adding another layer of complexity to gene expression and regulation (Figure 1).

**Figure 2.**
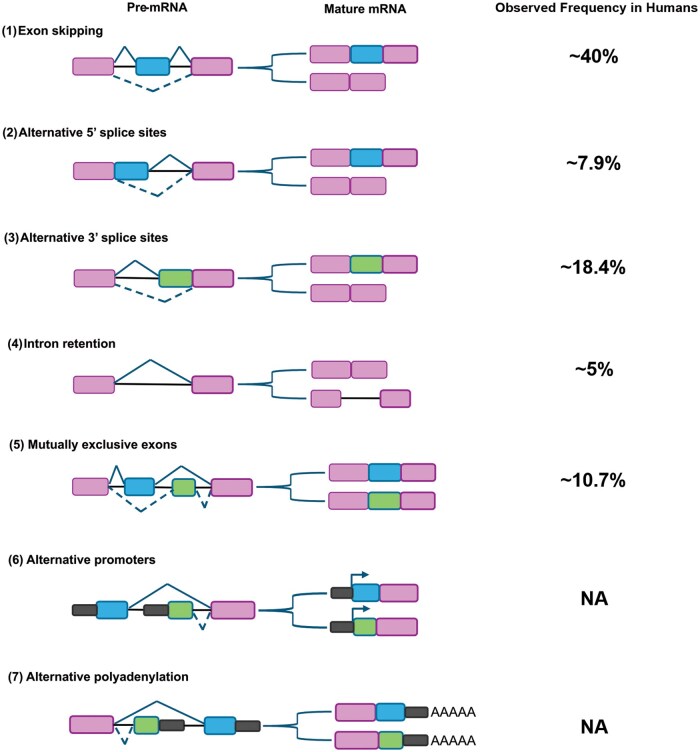
The principal types of alternative splicing of human genes. Pink boxes represent constitutively spliced exons, green boxes represent alternatively spliced exons, and introns are represented by lines. Bent lines indicate an alternative splicing event. Transcriptional promoters are indicated by solid arrows, and polyadenylation sites by the AAAAA sequence block. The figure illustrates the following splicing events: (**1**) exon skipping, (**2**) alternative 5′ splice sites, (**3**) alternative 3′ splice sites, (**4**) intron retention, (**5**) mutually exclusive exons, (**6**) alternative promoters, and (**7**) alternative polyadenylation.

Comprehensive analyses of alternative splicing in the human transcriptome have revealed that the vast majority of multiexon genes undergo alternative splicing. This process generates a substantial number of splicing events across major human tissues, with estimates indicating that approximately 95% of human genes containing multiple exons are subject to alternative splicing [22, 23]. To date, at least seven fundamental types of alternative splicing have been identified in eukaryotes. Exon skipping is the most common form observed in higher eukaryotes. The second most frequent type involves alternative 5' or 3' splice sites. Other forms of alternative splicing include intron retention, mutually exclusive exons, alternative promoter usage, and alternative polyadenylation (Figure 2) [18].

### Regulation of alternative splicing

The regulation of pre-mRNA splicing is predominantly orchestrated by the spliceosome, a sophisticated and dynamic ribonucleoprotein (RNP) complex. This large assembly comprises five small nuclear RNPs (snRNPs)–termed U1, U2, U4, U5, and U6–alongside a variety of auxiliary proteins that enhance the precision and efficiency of the splicing process. The spliceosome’s primary function is to accurately recognize splicing sites on pre-mRNA and catalyze the splicing reaction. In humans, the major spliceosome is responsible for processing more than 99.5% of introns, underscoring its critical role in gene expression regulation [24].

U1 and U2 snRNPs are responsible for recognizing and binding to the 5' and 3' splice sites of the intron on pre-mRNA. The U4/U5/U6 ternary complex is then recruited to bind to the pre-mRNA, inducing structural changes that circularize the originally linear pre-mRNA. This rearrangement brings the exons at both ends into close proximity. Subsequently, U1 and U4 are released, facilitating two transesterification reactions that result in intron cleavage and the successful joining of two exons [25]. Conversely, alternative splicing, which allows for variable exon inclusion, is primarily regulated by cis-acting regulatory elements recognized by RNA-binding proteins (RBPs). These proteins play crucial roles in RNA processing, maturation, localization, and translation [26]. Among these RBPs, serine-arginine (SR) proteins and heterogeneous nuclear ribonucleoproteins (hnRNPs) preferentially interact with intronic or exonic splicing enhancers or silencers (ISE/ISS or ESE/ESS), thereby influencing inclusion or exclusion of a constitutive exon [27]. Other RBPs, including members of the neuro-oncological ventral antigen (NOVA) and RNA-binding protein, fox-1 homolog (RBFOX) families, influence variable exon inclusion or skipping in a manner dependent on their position [28, 29].

### Alternative splicing and MAFLD

Alternative splicing is a fundamental mechanism that enhances protein diversity and has significant implications for the pathogenesis and progression of MAFLD [30]. It plays a vital role in regulating liver homeostasis, including circadian clock systems [31], liver growth, damage response, and regeneration [32, 33]. Additionally, it modulates the liver’s response to fluctuations in internal body temperature [34]. Growing evidence also indicates that disruptions in splicing processes, involving specific splicing factors and RBPs, are involved in the progression of MAFLD and related liver fibrosis [26]. These findings underscore the importance of understanding alternative splicing as a regulatory mechanism in liver function and disease progression.

#### Static versus adaptive or homeostatic RNA splicing in the liver

The liver plays a crucial role in maintaining metabolic homeostasis, utilizing both constitutive and adaptive regulation of RNA splicing [27].

The static regulation ensures the expression of essential genes involved in metabolism, detoxification, and protein synthesis in their correct forms. For instance, the SR-rich splicing factor (SRSF) 1 is vital for lipid homeostasis in hepatocytes; conversely, its inactivation can lead to steatosis and eventually liver failure [35]. RNA splicing is also pivotal during liver development, aiding in establishing key metabolic pathways early in life, notably, SRSF1 is crucial for genomic stability and global transcription control by mitigating the harmful effects of RNA-DNA hybrids (R-loops) [35]. SRSF5 is involved in the proliferation of liver cells during both development and the regeneration of the liver [36]. In addition, SRSF7 is significant for hepatocyte development, with higher expression levels in juvenile hepatocytes. Intriguingly, overexpression of SRSF7 can suppress cellular senescence *in vitro*, indicating its role in transcriptional regulation of hepatocyte maturation [37].

The adaptive functions of splicing in the liver reflect its ability to dynamically adjust splicing patterns in response to physiological changes, such as fasting, feeding, infection, and drug exposure. Splicing factors, including SR proteins (e.g. SRSF1, SRSF2) and hnRNPs (e.g. hnRNP A1, hnRNPL), are rhythmically expressed and regulated by the circadian clock. These factors influence the alternative splicing of clock-related genes, thereby linking RNA splicing to circadian regulation. Temporal changes in food availability also modulate hepatic splicing patterns [31]. For example, the RNA-binding protein non-POU domain-containing octamer-binding (NONO) responds to feeding signals by localizing to nuclear speckles, enhancing the rhythmic processing of glucokinase and glucose-transporter type 2 transcripts, which are crucial for glucose and lipid metabolism [38].

SFRS10/transformer-2 protein homolog beta (Tra2b) regulates adipocyte differentiation and low-density lipoprotein receptor splicing, whereas its deficiency has been associated with hepatic steatosis [39, 40]. Notably, heterogeneous nuclear ribonucleoprotein U(HnRNPU) downregulation worsens high-fat diet-induced MASH phenotypes by promoting alternative splicing of the tropomyosin receptor kinase B receptor [41]. Conversely, human antigen R (HuR) knockout impairs apolipoprotein B splicing, promoting the development of steatosis [42]. RBFOX2 deficiency results in an abnormal lipid distribution, which can be reversed by splice-switching oligonucleotides targeting scavenger receptor class B member 1 splicing [43]. In addition, downregulation of ubiquitin-specific protease 39–an essential factor in pre-mRNA splicing that maintains hepatic lipid balance by regulating alternative splicing in autophagy-related genes–results in steatosis and hepatotoxic effects [44]. Finally, the splice variant of transforming growth factor-β-activated kinase 1 (Tak1)–particularly Tak1-B when converted from Tak1-B to Tak1-A–has been associated with lipid infiltration [45].

#### Alternative splicing regulation in MAFLD progression

Alternative splicing is also implicated in MAFLD progression. During liver injury or inflammation, alternative splicing adapts by generating different isoforms of genes involved in apoptosis, fibrosis, and immune responses, aiming to control liver damage or stress [46, 47]. Conversely, alterations in this process can have deleterious consequences, exacerbating liver injury.

For example, alternative splicing of transcriptional enhanced associate domain 1 (TEAD1) produces an active isoform that promotes a fibrogenic response in hepatic stellate cells (HSCs)–which can be mitigated using antisense oligonucleotides (ASOs) [48]. Furthermore, Bcl-x is an antiapoptotic member of the *Bcl-2* gene family; the alternative splicing of Bcl-x yields isoforms with antagonistic roles in apoptosis. Notably, tipping the balance in favor of the pro-apoptotic isoform can induce HSC death, thereby presenting a promising therapeutic strategy for the treatment of fibrosis [49]. Kruppel-like factor 6 splicing produces variants linked to fibrosis severity and insulin resistance, with shorter isoforms being characterized by antifibrotic effects [50–52]. In addition, mitofusin 2 (MFN2) splicing generates isoforms that are critical for ER-mitochondria tethering, lipid metabolism, and fibrosis regulation [53, 54]. Interleukin 32 (IL32) alternative splicing produces isoforms (beta) that are involved in insulin resistance, endothelial dysfunction, hepatic fat accumulation, and fibrogenesis, whereas (gamma) isoforms are involved in inflammation [55–57].

SR proteins and hnRNPs, which play crucial roles in regulating alternative splicing, are implicated in the progression of MAFLD [58–60]. For example, conditional knockout of SRSF1 in the mouse liver results in a transient MASH-like phenotype, characterized by the concurrent development of steatosis, inflammation, and fibrosis [35]. Notably, this injury is ultimately resolved through a compensatory mechanism involving hepatocyte regeneration [35]. Recent studies have highlighted the critical roles of SR proteins in modulating liver disease progression. Specifically, SRSF3 has been shown to protect against fibrosis, steatosis, and carcinogenesis by regulating the splicing of genes, such as insulin-like growth factor 2 and the insulin receptor, as well as promoting lipid droplet clearance through lipophagy [61, 62]. Additionally, SRSF6 plays a key role in regulating the alternative splicing of genes associated with mitochondrial function. Hepatic deletion of death-associated protein kinase-related apoptosis-inducing kinase-2 (DRAK2) prevents the progression of hepatic steatosis to NASH by modulating RNA splicing. It directly interacts with SRSF6 and inhibits its phosphorylation by SRPK1, thereby influencing the splicing of mitochondrial function-related genes [63]. SRSF10 has been found to prevent premature intronic polyadenylation of metabolic genes, such as peroxisome proliferator-activated receptor alpha (PPARα), and disruptions in its function contribute to metabolic dysfunction and fibrosis, involving pathways like sirtuin1-SFRS10-lipin1 [64]. Finally, epithelial splicing regulatory protein 2 influences fibrosis progression in MAFLD by regulating epithelial-to-mesenchymal transition [33, 65].

## The bidirectional interaction between metabolism and alternative splicing

### Metabolic regulation of alternative splicing

Alternative splicing plays a crucial role in regulating cellular metabolism. Alterations in this process can contribute to the development of MAFLD. Conversely, metabolites can influence alternative splicing, and these changes can, in turn, modulate the expression of metabolic genes [66].

Glucose, a key energy source, regulates mRNA splicing through mechanisms involving the RNA helicase DExD-box helicase 21 (DDX21), which binds to RNA polymerase products during transcription. Glucose binding disrupts ATP-DDX21 interactions, causing the dissociation of DDX21 dimers and enabling its localization to the nucleoplasm–where it can interact with splicing factors to control alternative splicing [67]. Other RNA-binding proteins (RBPs), including leucyl-tRNA synthetase, also engage with glucose to regulate metabolic processes [68].

Furthermore, glucose may affect insulin mRNA splicing in mouse islets, facilitating insulin synthesis and secretion. In addition, complex oligosaccharides–including O-linked N-acetylglucosamine (GlcNAc)–are also involved in splicing regulation. GlcNAc, which is added to proteins in a glucose-dependent manner, alters splicing when O-GlcNAc transferase (OGT) is inhibited, affecting intron retention in OGT and related mRNAs [69]. Reduced O-GlcNAc modifications in turn increase intron splicing across mRNAs, potentially through changes in splicing factor phosphorylation and the modulation of liquid–liquid phase separation [70].

Beyond glucose, other energy sources may also affect RNA splicing. In this regard, fatty acids have the capacity to regulate the splicing of pre-mRNA encoding the glucagon-like peptide-1 receptor (GIPR) [71]. Under high-fat diet conditions, the splicing pattern of GIPR pre-mRNA undergoes modifications that lead to an increased proportion of active isoforms. This enhances β-cell sensitivity to GIPR, inducing high-fat diet-associated hyperinsulinemia [72]. Additionally, GIP promotes inflammation and insulin resistance in the adipose tissue of patients with obesity [73]. GIPR agonists increase lipolysis and favor insulin sensitivity, and the synergistic impact of GLP-1R-based therapies drugs targeting GIPR, has been reported [74].

Amino acids, another vital class of metabolites, operate as both signaling molecules and metabolic precursors. Recent research demonstrated that the key tryptophan metabolism enzyme indoleamine 2,3-dioxygenase 1 upregulates the amino acid transporter gene solute carrier family 1 member 5 (SLC1A5) and its splice variants, linking IDO-regulated splicing to intracellular amino acid levels [75]. SLC1A5 is a Na^+^-dependent transporter that specifically imports glutamine, with one splice variant functioning as a mitochondrial glutamine transporter. This suggests a potential crosstalk between mitochondrial metabolism and IDO-mediated alternative splicing [76, 77]. Additionally, Arginine–a conditionally essential amino acid–regulates immune responses by producing metabolites like proinflammatory nitric oxide and anti-inflammatory ornithine during inflammation [78].

The mechanistic target of rapamycin (mTOR) pathway, a central regulator of cellular metabolism, responds to small metabolites through specific receptors and in turn regulates RNA splicing [79]. Activation of the mTOR pathway can result in exon skipping, intron retention, and protein truncation [80]. In addition, it may modulate lipid metabolism via alternative splicing by phosphorylating the SR protein kinase (SRPK) 2, which enhances the splicing of mRNAs encoding lipogenic proteins. Interestingly, mTOR mRNA undergoes alternative splicing to generate the TORβ isoform, which preserves its catalytic activity. This highlights a reciprocal connection between mTOR signaling and the regulation of alternative splicing [81].

### Alternative splicing regulation of metabolic pathways implicated in MAFLD progression

Alternative splicing plays a pivotal role in regulating critical genes involved in metabolic pathways, including those governing insulin signaling, insulin resistance, and pancreatic beta cell function. These processes are intricately linked to the pathogenesis of MAFLD.

The insulin receptor (INSR), produced through the alternative splicing of the INSR gene, manifests in two forms: INSR-A and INSR-B. INSR-B helps protect beta cells from cell death induced by stress, whereas INSR-A makes them more susceptible to apoptosis [82]. Insulin modulates this form of alternative splicing via the rat sarcoma virus-mitogen-activated protein kinase (MAPK)/extracellular signal-regulated kinase signaling pathway, facilitating the inclusion of INSR exon 11 [83]. In addition, the splicing factor breast cancer amplified sequence 2 (BCAS2)–which is overexpressed in pancreatic beta cells–regulates beta cell function by promoting the aberrant splicing of genes, such as transcription factor 7-like 2, mouse double minute 2 (T*cf7l*2*)*, and synaptotagmin 7 (*Syt7*). These molecular events are believed to play a role in glucose intolerance and impaired insulin secretion [84]. Notably, cJUN N-terminal kinase (JNK)–a crucial mediator of insulin resistance–exacerbates metabolic disturbances by inhibiting the PPARα-fibroblast growth factor (FGF)-21 axis. JNK can also undergo alternative splicing to produce isoforms such as JNK2α and JNK2β, which exhibit distinct kinase activities toward different substrates, thus influencing liver metabolism and the development of insulin resistance [85, 86].

Alternative splicing is also crucial in regulating bile acid metabolism, particularly through proteins like farnesoid X receptor (FXR) and UDP-glucuronosyltransferase 2B4 (UGT2B4). FXR–a nuclear receptor involved in bile acid, lipid, and glucose metabolism–exists in multiple isoforms (FXRα1–8), each characterized by distinct functional properties [87, 88]. For instance, FXRα2 strongly induces the expression of the bile salt export pump, a bile acid transporter, while FXRα5 acts as a dominant-negative variant that disrupts normal FXR function, contributing to the onset of liver diseases and malignancies. The balance between FXRα isoforms is regulated by metabolic demands and can become dysregulated during inflammation, exacerbating bile acid toxicity [89]. Similarly, UGT2B4–which plays a role in bile acid glucuronidation–may also undergo alternative splicing to generate several isoforms with diverse regulatory roles in bile acid metabolism [90].

Alternative splicing is integral to lipid metabolism, particularly in genes associated with adipogenesis and hepatic lipid homeostasis [91, 92]. Systemic metabolic demands influence hepatic lipid metabolism by modulating the preference for specific FXRα isoforms through alternative splicing. These FXRα isoforms (FXRα1-4) regulate distinct target genes, allowing the liver to tailor its gene expression in response to metabolic signals [88]. PPARγ gene splicing, regulated by Staufen double-stranded RNA binding protein 1, governs adipogenesis, with PPARγ2 being especially relevant in fat tissue [93]. Additionally, alternative splicing of nuclear receptor corepressor (NCoR) generates isoforms (NCoRδ and NCoRω) that exert opposing effects on adipogenesis and liver lipid homeostasis [94].

**Figure 3.**
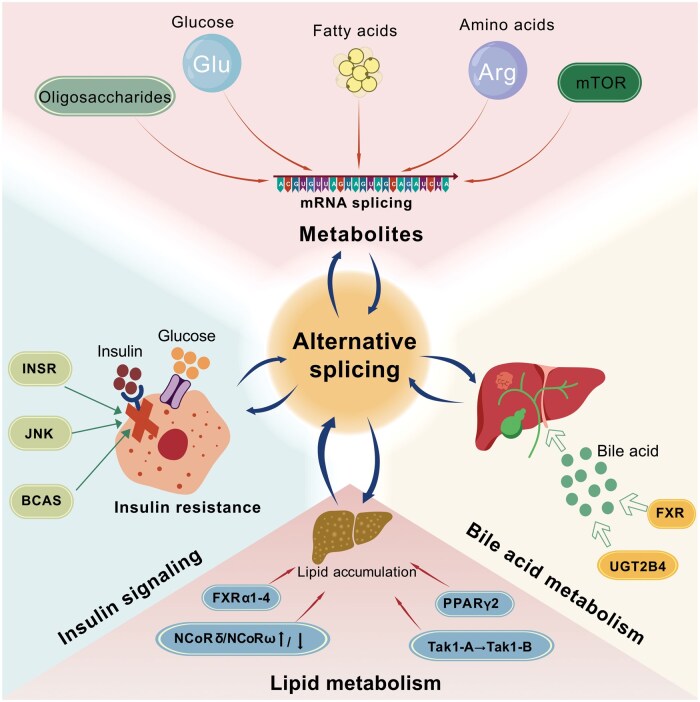
Interactions between metabolism and alternative splicing in the pathogenesis of MASLD.

In summary, alternative splicing modulates gene expression and protein function by interacting with metabolic signals, thereby influencing key processes involved in MAFLD, such as insulin resistance, bile acid metabolism, and lipid metabolism (Figure 3).

## Alternative splicing and epigenetic modifications in MAFLD progression

Alternative splicing is a fundamental process within the early stages of the ‘central dogma’ and is intricately regulated by epigenetic modifications, including histone modifications, DNA methylation, and the activity of long noncoding RNAs (lncRNAs). This dynamic, bidirectional interplay between alternative splicing and epigenetic mechanisms plays a crucial role in regulating key biological processes, such as lipid metabolism, inflammation, and fibrosis [95]. Histone modifications play a dynamic role in regulating transcription and significantly impact splicing by altering chromatin structure or signaling through epigenetic marks. Reversible histone acetylation modulates local chromatin architecture, facilitating transcriptional pauses that enhance the recognition of weak splice sites. Histone marks can recruit chromatin-remodeling proteins and splicing factors to transcription sites, thereby promoting co-transcriptional splicing. Acetylated histones enhance the processivity of RNA polymerase II and facilitate spliceosome assembly, ensuring efficient intron removal. Furthermore, chromatin-remodeling proteins can influence alternative exon inclusion or exclusion by recruiting specific splicing regulators [96, 97]. However, the mechanism of its action in MAFLD needs further investigation.

Furthermore, lncRNA can influence RNA splicing through direct interactions with splicing factors or by indirectly regulating their expression. A notable example is the metastasis-associated lung adenocarcinoma transcript 1 (MALAT1), an lncRNA highly expressed in adipose tissue, where it correlates positively with adipogenesis markers such as fatty acid-binding protein 4 and lipoprotein lipase. MALAT1 plays a critical role in transcriptional regulation of PPARγ, as well as in fatty acid metabolism and insulin signaling. Moreover, MALAT1 has been shown to modulate alternative splicing by interacting with splicing factors, thereby promoting specific splice variants that contribute to cancer progression. Notably, these variants exhibit anti-apoptotic properties, such as those associated with BIM and BIN1, and pro-proliferative characteristics, exemplified by TEAD1, highlighting the role of MALAT1 in promoting tumorigenesis through splicing regulation [98].

## Therapeutic modulation of alternative splicing

Advancements in understanding the role of alternative splicing in disease pathogenesis have underscored its potential as a therapeutic target. This has led to the possibility of exploring strategies to modulate splicing, including the use of small molecules and ASOs, for therapeutic intervention [99]. Currently, five globally approved drugs target RNA splicing, with most being ASO-based therapies, reflecting the growing clinical viability of splicing modulation as a treatment approach (Table 1) [100, 101].

**Table 1.** Globally approved drugs for splice-switching therapy

Drug	Disease	Target gene	Category	Company	Approval year
Nusinersen [129]	SMA	*SMN2*	ASO	Ionis Pharmaceuticals/Biogen	2016 (FDA)
Risdiplam [130]	SMA	*SMN2*	Small molecule	Roche/Genentech	2020 (FDA)
Eteplirsen [131]	DMD	*Dystrophin (DMD) Ex51*	ASO	Sarepta Therapeutics	2016(FDA)
Golodirsen [132]	DMD	*Dystrophin (DMD) Ex53*	ASO	Sarepta Therapeutics	2019 (FDA)
Viltolarsen [133]	DMD	*Dystrophin (DMD) Ex53*	ASO	NS Pharma, Inc.	2020 (FDA)

ASO, antisense oligonucleotide; CMV, cytomegalovirus; DMD, Duchenne’s muscular dystrophy; EMA, European Medicines Agency; FDA, Food and Drug Administration; HIV, human immunodeficiency virus; SMA, spinal muscular atrophy; SMN2 = survival motor neuron 2; TTR, transthyretin.

### Small molecules modulating RNA splicing

Recently, small organic molecules have gained attention as a promising strategy for modulating RNA splicing. These molecules can interact with various elements of the splicing machinery, including components of the spliceosome, trans-acting splicing factors, and RNA targets. They influence RNA processes by affecting RNA structure, metabolism, and degradation [102]. This approach offers a significant advantage in therapeutic convenience, as small molecules are orally bioavailable and can be administered without the need for specialized clinical settings. For example, pladienolide B–a small molecule inhibitor targeting the splicing factor 3b subunit 1 (SF3B1)–has demonstrated notable efficacy in modulating RNA splicing. It specifically inhibits SF3B1, disrupting splicing processes critical for tumor growth. Preclinical studies have shown that pladienolide B effectively suppresses the proliferation of HCC cells both *in vitro* and in mouse models, highlighting its potential as a therapeutic agent [103].

Furthermore, several small molecules targeting kinases involved in the phosphorylation of splicing factors, such as Serine-arginine (SR) protein kinases (SRPKs) and CDC2-like kinases (CLKs), have shown promising preclinical therapeutic potential for treating liver injury [104–106]. In the progression of MAFLD, abnormal splicing is regulated by SR proteins and hnRNPs, which are phosphorylated by CLKs, SRPKs, and other kinases activated by pathways like protein kinase B (Akt), phosphatidylinositol 3-kinase, and MAPK [107]. This suggests that these drugs might have potential in treating MAFLD. Although no splicing kinase inhibitors have yet reached clinical trials for liver diseases, a pan-CLK inhibitor is currently in phase 1 trials for solid tumors (NCT05084859). However, these inhibitors may affect multiple RBPs, which could reduce specificity and lead to potential off-target effects [27].

### Antisense oligonucleotides

Splice-modulating ASOs present a promising therapeutic strategy for correcting splicing errors and restoring the production of functional proteins. These short, single-stranded DNA molecules, typically consisting of 15–30 nucleotides, specifically bind to target mRNA sequences to either modulate splicing patterns or induce mRNA degradation. This precise interaction enables the correction of aberrant splicing events, thereby facilitating the synthesis of properly functional proteins [108]. ASOs can also block splicing enhancers (ESE/ISE) to prevent SR protein binding or inhibit splicing silencers (ESS/ISS) and promote exon inclusion by hindering hnRNPs. This innovative molecular strategy holds promise for treating various diseases. Importantly, ASOs exhibit unique absorption and metabolic pathways compared to conventional small molecules. They are predominantly administered via intravenous or subcutaneous routes and are chemically modified to enhance stability and protect against nuclease-mediated degradation [109].

ASOs are currently categorized into three generations based on their chemical modifications. The first generation utilizes a phosphorothioate backbone to confer nuclease resistance, albeit at the cost of reduced RNA affinity [110]. The second generation incorporates a 2′-alkyl modification, enhancing both affinity and nuclease resistance, although this may compromise overall efficacy [111]. The third generation features advanced chemical modifications, such as locked nucleic acids and phosphoramidate morpholino oligomers, which significantly improve target affinity and stability [112]. Furthermore, conjugating ASOs with ligands like N-acetyl galactosamine (GalNAc) facilitates liver-specific delivery via the asialoglycoprotein receptor, thereby enhancing pharmacokinetics [113]. Collectively, these modifications augment target affinity, nuclease resistance, biostability, and pharmacokinetics.

Concurrently, there have been substantial advancements in the clinical application of ASOs [100]. Notably, volanesorsen–a second-generation antisense oligonucleotide drug targeting and degrading the mRNA of apolipoprotein C-III–has been approved for treating genetic conditions that cause severe hypertriglyceridemia, such as familial chylomicronemia syndrome and familial partial lipodystrophy. Importantly, volanesorsen has demonstrated the ability to reduce hepatic fat fraction in three separate clinical trials [114], underscoring the therapeutic potential of ASO therapy for addressing steatosis. This advancement paves the way for innovative approaches to addressing splicing errors in MAFLD.

### Application of ASOs in MASH-associated fibrosis treatment

ASOs are emerging as a promising targeted therapy for MASH and its associated fibrosis [115], primarily through the regulation of specific genes [116]. In a recent study, the use of liver-targeted ASO to silence patatin-like phospholipase domain containing 3 (*PNPLA3*) in a knock-in mouse model with the human *PNPLA3* I148M variant resulted in reduced liver steatosis, inflammation, and fibrosis, alongside decreased inflammatory markers [117].

Additionally, ASOs targeting serine/threonine protein kinase 25 (Stk25) effectively reversed systemic hyperglycemia and hyperinsulinemia induced by a high-fat diet. This treatment improved glucose tolerance and insulin sensitivity while alleviating liver steatosis, metabolic steatohepatitis, and fibrosis in an obese mouse model [118]. Another study advanced this approach by utilizing GalNAc technology to develop hepatocyte-specific GalNAc-conjugated ASOs targeting Stk25. This method improved hepatic steatosis, inflammation, and fibrosis in mice exposed to chronic dietary lipids without any reported tolerability issues or systemic toxicity [119].

Similarly, research has demonstrated that deletion or knockdown of hepatic mitochondrial SH3BP5 (SAB; SH3 homology associated BTK binding protein) alleviates diet-induced metabolic syndrome, steatohepatitis, and hepatic fibrosis. Treatment with hepatocyte-targeted GalNAc-SAB-ASO successfully reversed steatohepatitis and fibrosis [120].

Several ASO-based therapies for treating MASH are currently in clinical trials (Table 2) [116]. For instance, AZD2693 (ION839), an ASO-GalNAc conjugate targeting the *PNPLA3* gene in patients with the I148M mutation linked to MASH, is under investigation in a Phase 2 trial [121]. In a Phase I trial, a siRNA targeting *PNPLA3* mRNA reduced hepatic fat accumulation in a dose-dependent manner in individuals with risk factors for MASH homozygous for the I148M variant, whereas it was not effective in heterozygous carriers [122, 123].

**Table 2.** ASO-based therapies against MASH currently in clinical trials

Drug	Targeted gene	Targeting agent	Phase	Trial identifier	Company
AZD2693 (ION839) [134]	*PNPLA3*	ASO/ASO-GalNAc conjugate	II	NCT04142424, NCT04483947	AstraZeneca and Ionis Pharmaceuticals
ALN-HSD [135]	*HSD17B13*	siRNA/(ESC+)-GalNAc conjugate	II	NCT05519475	Alnylam Pharmaceuticals
ARO-HSD [126]	*HSD17B13*	siRNA/siRNA-GalNAc conjugate	I	NCT04202354	Arrowhead Pharmaceuticals
AZD7503 (ION455) [127, 128]	*HSD17B13*	ASO/ASO-GalNAc conjugate	I	NCT05143905, NCT05560607	AstraZeneca and Ionis Pharmaceuticals
ION224 [136]	*DGAT2*	ASO/ASO-GalNAc conjugate	II	NCT04932512	Ionis Pharmaceuticals
BMS-986263 [137]	*HSP47*	retinoid-conjugated LNP containing siRNA	II	NCT03420768	Bristol Myers Squibb

*DGAT2*,  diacylglycerol acyltransferase 2; *HSD17B13*, 17 β-hydroxysteroid dehydrogenase 13; *HSP47*, heat shock protein 47; *PNPLA3*, patatin-like phospholipase domain-containing 3.

Other RNA interference drugs targeting hydroxysteroid 17-β dehydrogenase 13 (*HSD17B13*) are also being tested. The *HSD17B13* gene variant rs72613567 A-INS decreases the risk of MASH and liver damage [124]. ALN-HSD, a GalNAc-conjugated siRNA designed to silence *HSD17B13* expression, is being evaluated in a Phase 2 study (NCT05519475) [125]. Additionally, ARO-HSD (GSK4532990), a siRNA that completed Phase 1 trials (NCT04202354), showed good tolerance and reduced HSD17B13 expression and serum ALT levels in patients with MASH [126]. AZD7503 (ION455), a LICA ASO targeting *HSD17B13*, is under investigation in Phase 1 trials (NCT05143905 and NCT05560607) [127, 128]. Notably, the integration of ASOs with other therapeutic modalities has the potential to significantly enhance treatment outcomes.

## Conclusions and future perspectives

Alternative splicing is a critical post-transcriptional regulatory mechanism that significantly influences gene expression complexity and proteome diversity, particularly in the context of liver diseases. Dysregulation of alternative splicing, whether due to mutations in cis-regulatory elements or alterations in the activity of trans-acting splicing factors, plays a substantial role in the progression of fibrosis associated with MAFLD.

Elucidating the impact of these splicing abnormalities on key metabolic processes–including insulin resistance, lipid homeostasis, and bile acid metabolism–and their interaction with epigenetic modifications will provide essential insights into the pathogenesis of MAFLD-associated fibrosis. This understanding underscores the potential of targeting splicing mechanisms as a therapeutic strategy. Future research should prioritize identifying specific splicing events and regulatory networks that contribute to the transition from simple steatosis to more severe conditions like fibrosis and cirrhosis. The development of therapies that modulate splicing, including small molecules and ASOs, holds significant promise for precision medicine. These approaches aim to correct specific splicing defects, thereby reducing steatosis, inflammation, and fibrosis in targeted patient populations. Box 1 highlights some key areas for further research on alternative splicing in MAFLD. In summary, advancing our understanding of alternative splicing in MAFLD-associated fibrosis is crucial for the development of novel therapeutic interventions.

## Authors’ contributions

M.J. drafted the manuscript. S.A.A., W.-K.S., and Y.Y. conducted a critical review of the work and provided final approval. Z.P. and L.V. reviewed drafts and gave final approval of the work. M.E. conceived the project idea, critically reviewed drafts, and provided final approval of the work. All authors read and approved the final manuscript.
